# Supplementary material for: A Candidate Ac3-S-LPS Vaccine Against S. flexneri 1b, 2a, 3a, 6, and Y Activates Long-Lived Systemic and Mucosal Immune Responses in Healthy Volunteers: Results of an Open-Label, Randomized 2 Clinical Trial
Source: Vaccines (Basel). 2025 Feb 20;13(3):209. doi: 10.3390/vaccines13030209 (PMC11945589; doi:10.3390/vaccines13030209)
Supplement: Supplementary file 1 [file vaccines-13-00209-s001.zip › vaccines-3421928-supplementary.pdf]

Table S1. Dynamics of hematology parameters after single (V1) and repeated (V2) vaccination.

Time of blood collection	Red blood cells, 10^{12}/L	White blood cells, 10^9/L	Band neutrophils, %	Segmented neutrophils, %	Monocytes, %	Lymphocytes, %
**Normal values**	3.7 – 5.1	4.0 – 9.0	1 – 6	47 – 72	3 – 11	19 – 37
**Group 1 (dose 62.5 μg)**						
**Screening (n=40)**	4.48 ±0.36	7.04 ±1.25	1.20 ±0.65	61.33 ±2.40	4.98 ±1.14	32.43 ±1.96
**Day 2 after V1 (n=40)**	4.42 ±0.32	7.11 ±0.91	0.98 ±0.42	61.48 ±1.62	5.08 ±1.05	32.48 ±1.74
**Day 7 after V1 (n=40)**	4.45 ±0.32	6.94 ±0.70	0.98 ±0.42	61.33 ±1.75	5.40 ±1.26	32.20 ±1.67
**1 month after V1 (n=40)**	4.45 ±0.30	6.49 ±1.06	1.10 ±0.30	60.70 ±3.00	5.15 ±1.53	32.93 ±3.16
**Day 2 after V2 (n=40)**	4.45 ±0.30	6.56 ±0.81	1.10 ±0.30	61.15 ±1.97	5.10 ±1.24	32.55 ±2.05
**Day 7 after V2 (n=40)**	4.46 ±0.30	6.90 ±0.71	1.13 ±0.40	61.20 ±1.84	5.40 ±1.45	32.15 ±1.98
**1 month after V2 (n=40)**	4.42 ±0.32	6.66 ±0.79	1.00 ±0.39	61.63 ±2.64	5.15 ±1.21	31.95 ±2.48
**7 months after V2 (n=40)**	4.47 ±0.28	6.56 ±1.00	1.30 ±0.61	63.05 ±3.78	5.60 ±1.63	30.03 ±3.00
**Group 2 (dose 125 μg)**						
**Screening (n=40)**	4.39 ±0.35	6.97 ±1.41	1.25 ±0.49	61.85 ±2.79	4.73 ±1.28	32.25 ±2.46
**Day 2 after V1 (n=40)**	4.35 ±0.32	6.79 ±1.05	1.13 ±0.46	62.23 ±1.80	4.93 ±1.38	31.88 ±1.77
**Day 7 after V1 (n=40)**	4.36 ±0.31	6.70 ±0.79	1.03 ±0.36	61.15 ±1.48	5.05 ±1.52	32.68 ±1.87
**1 month after V1 (n=40)**	4.39 ±0.28	6.65 ±0.79	1.08 ±0.27	61.03 ±2.66	5.40 ±1.74	32.40 ±2.73
**Day 2 after V2 (n=40)**	4.42 ±0.30	6.80 ±0.86	1.00 ±0.39	61.18 ±1.93	5.00 ±1.34	32.75 ±2.49
**Day 7 after V2 (n=40)**	4.45 ±0.30	6.82 ±0.87	1.15 ±0.43	60.78 ±2.31	5.33 ±1.38	32.68 ±2.47
**1 month after V2 (n=40)**	4.48 ±0.30	6.77 ±0.88	1.00 ±0.32	61.20 ±1.95	5.28 ±1.38	32.45 ±1.92
**7 months after V2 (n=40)**	4.45 ±0.30	6.44 ±0.85	1.30 ±0.56	61.08 ±3.72	6.28 ±1.47	31.23 ±3.56

Data are presented as mean value ±SD.

Table S2. Dynamics of the concentration of hemoglobin in the red blood cells and erythrocyte sedimentation rate in male and female participants after single (V1) and repeated (V2) vaccination.

Time of blood collection	Male		Female	
	Hemoglobin, g/L	ESR, mm/h	Hemoglobin, g/L	ESR, mm/h
**Normal values**	130-170	2-10	120-155	2-15
**Group 1 (dose 62.5 μg)**				
**Screening**	153.75 ±15.2	5.25 ±1.89	139.83 ±10.87	11.33 ±4.95
**Day 2 after V1**	155.75 ±11.44	5.25 ±1.89	139.72 ±10.49	11.08 ±4.61
**Day 7 after V1**	155.50 ±11.45	5.25 ±1.89	139.69 ±9.88	9.44 ±3.19
**1 month after V1**	155.50 ±10.66	4.50 ±1.00	139.75 ±9.75	8.11 ±2.72
**Day 2 after V2**	155.50 ±11.12	5.00 ±1.41	139.78 ±9.73	8.19 ±2.72
**Day 7 after V2**	154.75 ±10.81	4.50 ±1.00	139.67 ±9.72	7.86 ±2.32
**1 month after V2**	154.75 ±10.50	4.00 ±0.82	139.78 ±9.56	7.47 ±2.12
**7 months after V2**	155.00 ±10.80	4.00 ±0.00	140.11 ±9.36	6.58 ±2.31
**Group 2 (dose 125 μg)**				
**Screening**	154.00 ±10.80	5.75 ±1.71	137.47 ±10.55	9.94 ±4.16
**Day 2 after V1**	154.00 ±10.86	5.75 ±1.71	137.61 ±10.3	9.53 ±3.87
**Day 7 after V1**	153.75 ±9.81	5.75 ±1.71	137.72 ±10.18	8.67 ±3.16
**1 month after V1**	151.25 ±7.37	4.50 ±1.73	137.81 ±9.68	8.06 ±2.9
**Day 2 after V2**	151.5 ±7.59	4.50 ±1.73	137.89 ±9.62	7.78 ±2.61
**Day 7 after V2**	153.00 ±8.29	4.50 ±1.91	137.94 ±9.65	7.83 ±2.68
**1 month after V2**	153.00 ±8.29	4.50 ±1.91	138.5 ±9.65	6.86 ±2.33
**7 months after V2**	153.25 ±7.97	4.25 ±1.5	138.44 ±8.97	6.08 ±1.83

Data are presented as mean value ±SD.

Table S3. Dynamics of blood biochemistry parameters after single (V1) and repeated (V2) vaccination.

Time of blood collection (n=40)	Creatinine, μmol/L	Glucose, mmol/L	Total bilirubin, μmol/L	ALT U/L	AST, U/L	Triglycerides, mmol/L	Cholesterol, mmol/L	HDL, mmol/L	LDL, mmol/L
**Normal values**	53-97	3.9-6.4	1.7-21	0-41	0-35	< 4.5	< 6.2	> 0.9	< 4.1
**Group 1 (dose 62.5 μg)**									
**Screening**	88.88±7.32	5.46±0.64	11.35±4.26	21.83 ±7.65	23.91 ±6.93	1.62±0.79	5.40±0.67	1.67±0.58	3.03±0.74
**Day 2 after V1**	88.68±7.18	5.41±0.61	11.67±3.87	21.59 ±7.61	23.61 ±6.45	1.64±0.77	5.38±0.67	1.69±0.58	3.01±0.73

**Day 7 after V1**	88.45± 7.08	5.41± 0.61	12.31± 3.32	21.24 ±7.17	22.93 ±6.30	1.66±0.75	5.40± 0.63	1.70± 0.58	2.98± 0.71
**1 month after V1**	88.19± 6.79	5.50± 0.64	11.29± 3.03	21.06 ±6.82	22.77 ±6.12	1.77±0.81	5.48± 0.59	1.71± 0.58	3.03± 0.72
**Day 2 after V2**	87.99± 6.66	5.50± 0.62	11.76± 2.62	20.97 ±6.78	22.70 ±6.17	1.82±0.87	5.46± 0.59	1.72± 0.57	3.01± 0.71
**Day 7 after V2**	87.79± 6.50	5.51± 0.60	12.09± 2.69	20.35 ±6.64	22.10 ±5.93	1.84±0.83	5.46± 0.57	1.71± 0.55	3.01± 0.70
**1 month after V2**	88.11± 6.37	5.54± 0.62	11.90± 2.23	20.70 ±6.60	22.38 ±5.92	1.86±0.82	5.43± 0.61	1.73± 0.55	2.98± 0.69
**7 months after V2**	88.12± 6.29	5.56± 0.60	11.57± 2.55	20.23 ±6.17	21.72 ±5.73	1.89±0.81	5.48± 0.58	1.75± 0.55	3.00± 0.62
**Group 2 (dose 125 μg)**									
**Screening**	86.28± 8.86	5.64± 0.55	10.49± 4.11	22.72 ±8.55	21.96 ±6.38	1.63±0.78	5.26± 0.75	1.54± 0.26	3.01± 0.68
**Day 2 after V1**	86.13± 8.82	5.65± 0.58	11.14± 3.78	22.29 ±8.44	22.25 ±5.49	1.63±0.76	5.30± 0.65	1.53± 0.27	3.01± 0.66
**Day 7 after V1**	85.68± 8.69	5.63± 0.57	11.60± 3.14	22.32 ±8.15	22.22 ±5.68	1.66±0.82	5.26± 0.67	1.56± 0.27	3.02± 0.66
**1 month after V1**	85.96± 8.06	5.66± 0.56	10.73± 3.53	22.48 ±8.37	22.06 ±5.49	1.80±0.97	5.31± 0.64	1.55± 0.25	3.04± 0.61
**Day 2 after V2**	85.89± 8.16	5.66± 0.52	11.18± 3.24	22.52 ±8.24	22.43 ±5.54	1.81±0.97	5.32± 0.63	1.55± 0.25	3.05± 0.59
**Day 7 after V2**	85.95± 8.12	5.67± 0.55	10.99± 2.95	21.94 ±8.09	22.08 ±5.42	1.81±0.96	5.33± 0.63	1.54± 0.27	2.97± 0.65
**1 month after V2**	85.82± 7.85	5.69± 0.56	10.93± 2.76	21.74 ±7.76	22.07 ±5.06	1.83±0.94	5.32± 0.63	1.57± 0.26	3.00± 0.66
**7 months after V2**	86.28± 8.02	5.68± 0.56	11.76± 2.37	21.46 ±7.39	21.75 ±4.62	1.86±0.94	5.33± 0.63	1.60± 0.27	3.02± 0.64

Data are presented as mean value ±SD.

Table S4. Dynamics of urinalysis parameters after single (V1) and repeated (V2) vaccination.

Group/ Time of urine collection	pH	Specific gravity, g/mL	Protein, g/L	Glucose, mmol/L
**Normal values**	5.0-6.5	1.005-1.030	negative	negative
**Group 1 (dose 62.5 μg)**				
**Screening (n=40)**	5.5 (5.5 – 6)	1.020 (1.015 – 1.025)	negative	negative
**Day 2 after V1 (n=40)**	5.5 (5.5 – 6)	1.020 (1.015 – 1.025)	negative	negative
**Day 7 after V1 (n=40)**	6 (5.5 – 6.38)	1.020 (1.015 – 1.025)	negative	negative
**1 month after V1 (n=40)**	5.5 (5.5 – 6)	1.025 (1.025)	negative	negative
**Day 2 after V2 (n=40)**	5.5 (5.5 – 6)	1.025 (1.025)	negative	negative
**Day 7 after V2 (n=40)**	5.5 (5.5 – 6.38)	1.025 (1.025)	negative	negative
**1 month after V2 (n=40)**	5.5 (5.5 – 6)	1.025 (1.025)	negative	negative
**7 months after V2 (n=40)**	5.5 (5.5 – 6)	1025 (1.025)	negative	negative
**Group 2 (dose 125 μg)**				

**Screening (n=40)**	5.5 (5.5 – 6)	1025 (1015 – 1025)	negative	negative
**Day 2 after V1 (n=40)**	5.5 (5.5 – 6)	1025 (1020 – 1025)	negative	negative
**Day 7 after V1 (n=40)**	5.5 (5.5 – 6)	1025 (1015 – 1025)	negative	negative
**1 month after V1 (n=40)**	5.5 (5.5 – 6)	1025 (1025)	negative	negative
**Day 2 after V2 (n=40)**	5.5 (5.5 – 6)	1025 (1025)	negative	negative
**Day 7 after V2 (n=40)**	5.5 (5.5 – 6)	1025 (1025)	negative	negative
**1 month after V2 (n=40)**	5.5 (5.5 – 6)	1025 (1025)	negative	negative
**7 months after V2 (n=40)**	5.5 (5.5 – 6)	1025 (1025)	negative	negative

Data are presented as median (interquartile range)
